# Meetings of Societies

**Published:** 1947

**Authors:** 


### Meetings of Societies

### Bristol Medico-Chirurgical Society

The seventh meeting of this Society was held on Wednesday, April 9th. Professor Alan Moncrieff gave a stimulating address entitled "**Child Health.**" He recounted some of the failures in this field as seen in this country of late years, giving it as his opinion that inadequacy in training in Paediatrics and the failure of co-operation between Public Health and Hospital sides of the service had been largely responsible. He recounted improvements which had occurred in various cities of the country during the past few years, and appealed for further co-operation and for better educational facilities in the future.

The eighth meeting was held on Wednesday, May 14th. A combined paper entitled "**The Rhesus Factor**" was then delivered by Dr. B. Corner and Dr. Tovey. The latter discussed the pathological factors involved and the former the clinical aspects and methods of treatment. The results of upward of fifty local cases were given.

